# Profile of oropharyngeal swallowing in healthy Brazilian adults and older adults

**DOI:** 10.1016/j.bjorl.2024.101494

**Published:** 2024-08-31

**Authors:** Giovana Piovesan Dall'Oglio, Eliézia Helena De Lima Alvarenga, Leonardo Haddad, Mateus Morais Aires, Márcio Abrahão

**Affiliations:** a Universidade Federal de São Paulo (UNIFESP), Departamento de Otorrinolaringologia e Cirurgia de Cabeça e Pescoço, São Paulo, SP, Brazil; b Universidade de Pernambuco (UPE), Faculdade de Ciências Médicas, Recife, PE, Brazil; c Universidade Federal de Pernambuco (UFPE), Hospital das Clínicas, Recife, PE, Brazil

**Keywords:** Deglutition disorders, Fiberoptic endoscopic evaluation of swallowing, Swallowing physiology, Age factors, Dysphagia

## Abstract

**Objectives:**

To describe the findings of Fiberoptic Endoscopic Examination of Swallowing (FEES) in asymptomatic young and older adults, comparing results across different age groups. Additionally, this study aims to test the Eating Assessment Tool (EAT-10) as an instrument to identify dysphagia risk.

**Methods:**

A prospective cross-sectional observational analysis was conducted on a sample of individuals aged 20 and above, asymptomatic for dysphagia, stratified by age groups. The EAT-10 questionnaire was completed, and the FEES was employed to assess oropharyngeal swallowing function. Various parameters, including salivary stasis, swallowing reflex trigger, swallowing sequence, residue, penetration, and aspiration were blindly analyzed by two otolaryngologists.

**Results:**

A total of 184 participants were included, with a mean age of 44.7 ± 18.5 years. There was good to excellent agreement between examiners for FEES parameters. The EAT-10 score ≥3 suggested dysphagia risk in 7.6% (n = 14) of the sample, with no association with age or any FEES parameter. Individuals aged ≥80 years presented more residue (50%; n = 5/10) compared to younger individuals (11.5%; n = 20/174; *p* =  0.039). Salivary stasis was found exclusively in individuals aged ≥60 years (n = 5/39; 12.8%; *p* = 0.027). Age did not influence on the swallowing reflex trigger, swallowing sequence, penetration, and aspiration. Penetration was observed in 4.9% (n = 9) of subjects and aspiration occurred in 0.5% (n = 1) of subjects, with no statistical significance in age groups.

**Conclusion:**

Age does not have a linear influence on swallowing in healthy adults and elderly people. However, individuals aged ≥80 years showed a higher prevalence of residue, and individuals aged ≥60 years showed a higher prevalence of salivary stasis, suggesting an increased risk or presence of dysphagia. Other FEES parameters were not influenced by age. These findings provide valuable insights into the nuanced dynamics of swallowing across different age groups, emphasizing the importance of age-specific considerations in dysphagia assessment.

**Level of evidence:**

4.

## Highlights

• Age ≥80 years linked to higher salivary stasis and residue.
• FEES reveals no age impact on penetration and aspiration.
• Number of swallows to clear bolus was two during lifespan.
• The swallowing reflex was triggered up to the vallecula in 99% in all consistencies.
• Presence of residue and penetration indicate impaired swallowing efficiency and safety.

## Introduction

Oropharyngeal swallowing is a complex sensorimotor mechanism involving multiple head and neck muscles, various cranial nerves, and the spinal roots of the first three cervical nerves. Its execution relies on the coordination and interaction of the central nervous system through cortical, sub-cortical, and brainstem structures, where the swallowing centers are located.^1^ Coordination between breathing and swallowing is necessary to reconfigure the respiratory tract into a digestive pathway, ensuring the protection of the airways. This coordination facilitates the safe transfer of food from the mouth to the stomach, resulting in hydration, nutritional gain, and pleasure in eating, crucial for maintaining a high quality of life.^1^, ^2^

Dysphagia is the difficulty in swallowing food or pills and in managing saliva, bile, nasal, oral, and tracheobronchial secretions.^3^ It can occur at any stage of swallowing.^1^, ^2^, ^3^ The prevalence of dysphagia in the adult population ranges from 2.3% to 22.0%, often being underestimated.^3^, ^4^ The global aging of the population, associated with an increased prevalence of dysphagia, has the potential to become a significant public health concern.^5^, ^6^

The Fiberoptic Endoscopic Examination of Swallowing Safety (FEES), described in 1988 by Langmore, Kenneth, and Olsen,^5^ evaluates the pharyngeal phase of swallowing and, akin to videofluoroscopy (VFS), is considered the gold standard for assessing swallowing. In Brazil, FEES is referred to as Videoendoscopia da Deglutição (VED). FEES enables direct, structural, and functional assessment with image capture, inferring the pathophysiology of dysphagia. It also establishes the safety of swallowing and guides rehabilitation. The portability of the equipment facilitates assessments in different locations, using patients' usual food. Moreover, it offers an indirect perception of the oral and esophageal phases of swallowing, has no radiation exposure, and can be repeated several times throughout treatment, making it the instrument of choice.^7^

In the literature, there is a knowledge gap regarding the swallowing mechanisms in healthy adults and older adults, leading to conceptual divergence about the healthy aging of swallowing (presbyphagia). This divergence impacts the accurate estimation of dysphagia prevalence. There is no consensus on whether the swallowing mechanism in adults and the elderly changes over the lifespan, how it changes, or when these changes occur, making it even more difficult to diagnose dysphagia. In Brazil, there is a lack of epidemiological studies on dysphagia prevalence in the healthy population and on normal swallowing patterns. Therefore, there is a pressing need to determine the profile of swallowing in healthy adults and older adults in our country.

The objective of this study is to describe the findings of the FEES in asymptomatic young and older adults, comparing these findings across different age groups. Additionally, the study aims to establish standards of normal swallowing in the Brazilian population and to test the Eating Assessment Tool (EAT-10) as an instrument to identify the risk of dysphagia, thereby contributing to the consolidation of diagnostic criteria for dysphagia.

## Methods

This was a cross-sectional observational study, approved by the Research Ethics Committee of Hospital São Paulo-University Hospital/UNIFESP (CoEP) approved it under letter no. 577/2019. All participants provided informed consent before participating in the study.

The study population comprised individuals aged 20 and above with no prior complaints or history of dysphagia. Recruitment occurred at the Otorhinolaryngology and Geriatrics outpatient clinics at the UNIFESP, including students, staff, doctors, and companions. Additional participants were recruited from a social gatherings (Santo Amaro) and directly from the homes of some super elderly individuals (≥80-years). Exclusion criteria encompassed subjects with neurological diseases, including declared dementia, or a history of chemo/radiotherapy, head and neck surgery and prolonged orotracheal intubation (≥48-hs).

The sample size calculation was derived from age data of the most recent local population census, and from a previous publication by our team, in which 39% of individuals aged ≥65-years exhibited residue in FEES.^8^ Assuming a baseline percentage of 20% for residue in the normal population of all ages, with a 95% Confidence Interval and 6% precision, the sample consisted of 184 subjects, proportionally distributed according to the adult population of the city of São Paulo across different age groups. Individuals aged above 60 years were considered older adults, and those above 80 years were considered super-older adults.

Participants initially completed the EAT-10 questionnaire, with a score ≥3 being suggestive of risk of dysphagia.^9^, ^10^ FEES was performed according to the technique described by Langmore^7^ with modifications, with a 3.2 mm diameter nasofibroscope (Machida Flexible Scope, Machida Inc., Orangeburg NY, USA), connected to an image capture and visualization system (Telepack Storz, Tuttlingen, Germany).

The nasofibroscope was introduced through the widest nasal fossa of the subject while awake, in a sitting position mimicking the posture during eating, without topical anesthetic. We noticed velopharyngeal function, anatomical aspect of the larynx and salivary stasis, defined as the presence of pre-swallowing secretion and/or saliva in the pharynx. A spontaneous sip of water from a 50 mL disposable cup was offered for training and understanding of the procedure, which was disregarded for the exams results.

Functional assessments included various consistencies such as liquid, thickened liquid, pasty, and solid. The water was thickened with the Resource Thicken up Clear (Nestlé Health Science, São Paulo, Brazil), according to the mixture described by the manufacturer. The pasty consistency was stained green and the liquid and thickened liquid consistencies were stained blue with food coloring. The solid used was salt crackers, without coloring. The food was offered to the volunteer through a 50 mL disposable cup so that they could spontaneously ingest it without verbal orders or cues, as many times as necessary until they felt a clear throat.

We started by offering the pasty consistency, followed by thickened liquid, thin liquid and solid. The following parameters were recorded for each offering:^11^, ^12^, ^13^

1 Posterior spillage: Presence of the food bolus beyond the oral cavity in a posterior direction without swallowing movement.
2 Swallowing reflex trigger: Anatomical site where the swallowing movement is triggered, considered delayed when it occurs posterior to the vallecula (example: piriform sinus).
3 White-out: Flash seen on the endoscopic image during the pharyngeal contraction of swallowing.
4 Residue: The presence of food residue after the first spontaneous swallow, through the sensation of complete swallowing by the subject. It was classified as: nothing (no residue), trace (some trace was observed) and more than trace (some volume was observed in addition to the trace).
5 Swallowing sequence: Swallows that occurred spontaneously after the first swallow.
6 Number of swallows: Spontaneous swallows required to completely clear the bolus.
7 Esophago-Pharyngeal Reflux (EPR): Return of swallowed contents to the larynx after swallowing.
8 Laryngeal penetration: Presence of saliva or food residue in the laryngeal vestibule (from the laryngeal face of the epiglottis to the upper edge of the Vocal Folds (VF).
9 Laryngotracheal aspiration: Presence of saliva or food residue below the level of the VF.
10 Protective reflex: The subject's response to penetration and/or aspiration. This behavior can manifest as efficient coughing (successful removal of contents from the airway), inefficient coughing (contents remain in the airway), VF adduction, or absence of protective movements (silent aspiration).

Lastly, we perform the provocation test by offering 90 mL of water based on the Yale test.^14^ Failed is considered inability to take the total volume uninterruptedly, the presence of a wet voice or coughing within one minute of the offer.^14^

The recordings, randomized and blinded to the subject's age, were independently analyzed by two otorhinolaryngologists with over 15 years of experience in performing and interpreting FEES. In case of disagreement, the examiner reviewed the videos to determine the final result.

### Statistical analysis

Qualitative characteristics were described using absolute and relative frequencies, and agreement between examiners was assessed using the Kappa coefficient and Kendall's Taub-b coefficient. The Chi-Square test was employed to analyze associations between the presence of residue and other variables. Fisher's exact test assessed qualitative parameters of FEES, and correlations with EAT-10 were examined using Chi-Square or likelihood ratio tests. Odds ratios with 95% Confidence Intervals were calculated using bivariate logistic regressions to estimate associations between complaints and findings. Statistical analyses were conducted using IBM-SPSS for Windows software version 22.0, with tabulation performed using Microsoft Excel 2010 software. Tests were conducted at a significance level of 5%.

## Results

The sample comprised 184 subjects, with 53.3% being female, and the average age was 44.7 ± 18.5 years (ranging from 20 to 97 years). Table 1 displays the distribution by age group. The Kappa coefficient of agreement between the examiners was classified as good to excellent, ranging from 0.65 for the presence of salivary stasis, 0.76 for laryngeal structural findings, 0.79 for the presence of penetration and 1.0 for triggering the swallowing reflex and aspiration.

**Table 1:** Number of patients according to age.

Age (years)	n (%)
20 to 29	44 (23.9%)
30 to 39	40 (21.8%)
40 to 49	35 (19.0%)
50 to 59	26 (14.1%)
60 to 69	18 (9.8%)
70 to 79	11 (6.0%)
≥ 80	10 (5.4%)
Total	184 (100%)

The EAT-10 score was ≥3 in 7.6% (n = 14) of the sample. There was no association between the EAT-10 score and any parameter assessed by FEES, nor with age (*p* = 0.80; *p* = 0.95, respectively).

All subjects exhibited normal velopharyngeal function, while structural changes in the VF were identified in five individuals (2.7%), including hyperemia, vasculodysgenesis, leukoplakia, and edema (n = 2).

Salivary stasis was identified in five subjects (2.7%), three were ≥60 years and two were ≥80 years, with a statistically significant increase in salivary stasis as age increased (*p* = 0.027).

Posterior spillage was not observed. The swallowing reflex was triggered up to the vallecula for the entire sample, except for one subject (0.5%) in the fourth decade, for the pasty consistency. It occurred in the piriform sinus and was considered delayed, but without statistical significance (*p* = 0.97). White-out occurred in 100% of the sample. Residue was identified in 25 subjects (13.6%) and 37 offerings (5.0%). Two subjects presented residue for two consistencies, and five presented residues for three consistencies tested.

Table 2 displays the distribution of residue presence according to age group, indicating a statistically significant association with age, with a higher proportion of residue in individuals aged ≥80 years (*p* = 0.039). There was also an association between the presence of residue and an increase in the number of swallows in all the consistencies tested (*p* < 0.001).

**Table 2:** Prevalence of residue according to age.

Age (years)	n (%)
20 to 29	4 (9.1%)
30 to 39	6 (15.0%)
40 to 49	3 (8.6%)
50 to 59	3 (11.5%)
60 to 69	3 (16.7%)
70 to 79	1 (9.1%)
≥80	5 (50.0%)
Total	25 (13.6%)

In the liquid consistency, 100% of subjects had up to two swallows for pharyngeal clearing, 90.8% for the thickened liquid consistency, 94.6% for the pasty consistency and 95.1% for the solid. Up to three swallows were observed in 95.7% of subjects for the thickened liquid consistency, 97.3% for the pasty consistency and 97.8% for the solid. Presence of 3 swallows for pharyngeal clearing was identified in 31 subjects (16.8%), considering at least one consistency, distributed according to age group as follows: second, third and seventh decades (n = 5 in each decade), fourth and sixth decades (n = 6 in each decade); fifth and eighth decade (n = 2 in each decade); with no statistical significance in terms of age group.

Nine subjects had penetration (4.9%), distributed according to age group as follows: second, sixth and seventh decades (n = 1 in each decade), fourth decade (n = 4) and eighth decade (n = 2); with no statistical significance in terms of age group. One subject (0.5%) in the sixth decade experienced aspiration for solid consistency, with concomitant penetration for solid consistency. All the subjects who experienced penetration and/or aspiration had an efficient laryngeal protection reflex.

**Fig. 1:**
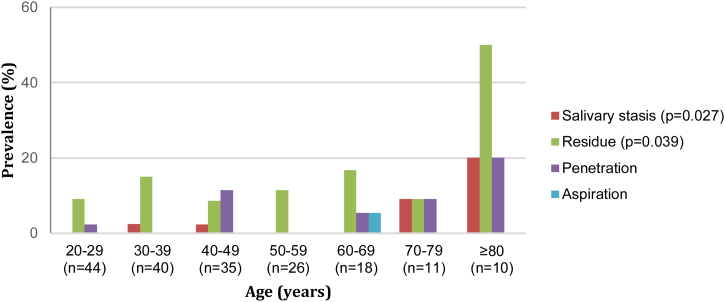
Prevalence of FEES findings according to age.

Fig. 1 provides a visual description of FEES findings according to age group, highlighting a higher proportion of findings in individuals aged ≥80 years.

Seventeen individuals (8.7%) failed the Yale test due to fluid fractionation. There was no association between the FEES findings, except for penetration and residue (*p* = 0.003). We do not observe EPR in any consistency offered.

## Discussion

This study provides a detailed description of the findings of the FEES in a sample of 184 asymptomatic young and older adults, with a focus on the impact of aging on various swallowing parameters. Age-related changes, known as presbyphagia, may affect the swallowing function, potentially leading to dysphagia. However, distinguishing between normal aging and pathological dysphagia is challenging.

Humbert and Robbins^15^ raised the question of when a change in swallowing pattern becomes pathological. Our findings among the super elderly suggest an increased likelihood of presbyphagia evolving into dysphagia. We argue that the presence of residue and penetration indicates compromised swallowing efficiency and safety, hence indicating dysphagia.

The presence of salivary stasis was predominantly observed in older adults, with a significant increase in salivary stasis in those aged 60 years and above. Residue presence was significantly higher in individuals aged 80 years and above, suggesting that older adults are more likely to have inefficient bolus clearance, which can increase the risk of aspiration and other complications. The association between residue and an increased number of swallows across all consistencies further emphasizes the impact of aging on the efficiency of the swallowing process.

In a previous study^8^ involving healthy older adults (≥60-years), we identified residue in 39% of the sample, penetration in 9%, salivary stasis in 6%, and aspiration in 2%. In the current study, we observed residue in 23% of subjects aged ≥60-years, penetration in 10%, salivary stasis in 8% (indicating difficulty in managing their own secretions, especially among the super elderly ≥80-years-old), and aspiration in 2%. The difference in residue findings is attributed to the use of a milk product with a pasty consistency in the first study, leading to difficulty in clearance.

Penetration was observed in 4.9% of subjects, with no significant age-related distribution, and aspiration was rare, occurring in only one individual. Importantly, all subjects with penetration or aspiration exhibited an effective laryngeal protection reflex, indicating a preserved compensatory mechanism in asymptomatic individuals. This finding is critical as it suggests that the presence of penetration or aspiration alone, without symptoms, may not necessarily indicate a pathological condition but rather a normal variation within a healthy aging population. Indeed, it has been suggested that these phenomena can be observed in a small percentage of healthy adults by VFS or FEES, more frequently in healthy older adults.^16^

We need to better understand the spontaneous sequencing of swallowing. We observed that 3.8% of individuals did not show sequencing for one or more consistencies, particularly in sixth decade (22%). This result may aid in interpreting the pathophysiology of presbyphagia, suggesting that non-sequencing could precede the presence of residue or trigger compensations to avoid residue. We suggest another possibility, that the involvement of the central pattern generator as a compensation may require greater pharyngeal stimulation (varying volumes and consistencies) for subsequent oropharyngeal swallowing occurs for clearing, considering the significant decline in systemic neuromuscular function and performance associated with aging.^17^

We concur with Santoro et al.^18^ that normal oral containment implies the absence of anterior and posterior spillage. In our cohort of asymptomatic individuals, the triggering of the swallowing reflex occurred up to the vallecula in all patients. This finding reinforces that normal swallowing occurs with the reflex trigger in this region. A delay in this trigger should indicate dysphagia.

The EAT-10 score ≥3, suggestive of dysphagia risk, was found in 7.6% of the sample. However, unlike other authors,^19^, ^20^ we found no association between EAT-10 and any FEES parameter or age. This lack of correlation highlights the need for comprehensive diagnostic approaches that combine subjective symptom questionnaires with objective FEES assessments to accurately diagnose dysphagia. The EAT-10 should be regarded as a screening instrument for the instrumental assessment of swallowing rather than a diagnostic tool for dysphagia. Additionally, in line with expectations for a healthy population, we observed no association between EAT-10 scores and age groups.

There has been an increase in the maximum age considered in research over time, suggesting that researchers are exploring the aging of the population.^21^, ^22^ Our findings highlight several significant associations between age and specific aspects of swallowing function, which are crucial for understanding the nuances of presbyphagia and distinguishing it from pathological dysphagia.^21^, ^23^ This limitation in our work and in the literature underscores the need to include more super elderly in clinical research to ascertain whether they are indeed a higher-risk group for dysphagia, as our findings suggest. Further research is essential to pinpoint the age continuum at which observed changes in swallowing begin to emerge.

## Conclusions

The swallowing profile in the healthy adults and eldery population was described. The age factor influences the physiology of swallowing in individuals asymptomatic for dysphagia, as assessed by FEES. Elderly individuals above 80 years old exhibited a higher prevalence of salivary stasis and residue, indicating an increased risk or presence of dysphagia. The presence of residue was associated with penetration and indicates altered swallowing efficiency, therefore dysphagia.

There was no age-related influence on other assessed parameters, such as swallow initiation, swallow frequency, penetration, or aspiration. Notably, all subjects experiencing penetration and/or aspiration demonstrated an effective laryngeal protection reflex. The EAT-10 questionnaire was not a valid tool to evaluate subjects with no prior complaints or history of dysphagia. These conclusions contribute valuable insights into the nuanced dynamics of swallowing across different age groups and emphasize the significance of considering age-specific factors in the assessment of dysphagia.

## Funding

The authors have no funding or financial relationships to disclose.

## Conflicts of interest

The authors declare no conflicts of interest.
